# Climate anxiety, coping strategies and planning for the future in environmental degree students in the UK

**DOI:** 10.3389/fpsyg.2023.1126031

**Published:** 2023-07-26

**Authors:** Cami Daeninck, Vasiliki Kioupi, Ans Vercammen

**Affiliations:** ^1^Centre for Environmental Policy, Imperial College London, London, United Kingdom; ^2^Institute of Education, University College London, London, United Kingdom; ^3^The School of Communication and Arts, The University of Queensland, St. Lucia, QLD, Australia

**Keywords:** climate change, eco-anxiety, environmental education, mental health, well-being, career decision-making, ecopsychology

## Abstract

**Introduction:**

There is increasing recognition of the mental health burden of climate change and the effects on general well-being, even in those who have not (yet) experienced direct impacts. Climate anxiety, which is prominent among young people in particular, describes a state of heightened distress about the (future) effects of climate change. Despite evidence of a link between engagement in climate change issues and heightened climate anxiety, there is a dearth of knowledge on how this affects emerging professionals preparing for careers in the environmental sector. Furthermore, there is a paucity of literature regarding the extent to which young adults are coping with their thoughts and feelings about climate change, and the extent to which they consider climate change in making future plans.

**Methods:**

The aim of this study was to understand the occurrence and personal management of climate anxiety in UK university students through an online questionnaire. This study was the first to investigate the association between climate anxiety, coping strategies and future planning in university students.

**Results and discussion:**

Environmental degree students (*n* = 249) reported greater levels of climate anxiety, more frequent employment of all three examined coping strategies and in particular considered climate change as a factor in their career plans, as compared to their non-environmental degree counterparts (*n* = 224). Problem-focused coping was the most commonly endorsed strategy, although the prior literature on coping suggests that this may not be sustainable for individually intractable problems. Highly climate-anxious students were more likely to consider climate change in all five decision-making domains, including family planning, long-term habitation, career, financial and travel decisions. This study has identified a need to communicate effective climate anxiety coping strategies to environmental practitioners, university students and educators. Additional research is required to validate the study findings and investigate what motivates students to incorporate climate change into future plans.

## 1. Introduction

Anthropogenic climate change continues to transform the planet at an alarming rate, with the environmental and social consequences to species and ecosystems becoming more apparent each year (IPCC, 2022). Climate change is now recognized as one of the greatest challenges of the 21st century (Ágoston et al., 2022), and empirical evidence supports a relationship between climate change and mental health (Charlson et al., 2021). The effects of climate change on mental health are diverse and include both direct and indirect impacts. Direct impacts of climate change involve first-hand exposure to geophysical changes, such as more frequent and extreme weather events. A growing body of research shows that experiencing such events raises the risk of mental health disorders including post-traumatic stress disorder, sleep disruption, depression or low mood and even suicidal ideation (see Hayes et al., 2018; Cianconi et al., 2020; and Charlson et al., 2021 for recent reviews). Damage to infrastructure and supply chains can also disrupt healthcare provision, resulting in a situation of increasing demand for services, with dwindling capacity for the system to cope and respond (Lawrance et al., 2021). In addition, indirect impacts of climate change include psychological and emotional repercussions that result from the idea of climate change, uncertainty about the future and anticipated impacts, observing the loss experienced by others and witnessing challenges occurring elsewhere in the world (Ojala et al., 2021).

Various terms encompass the emotional responses to climate change, including ecological grief, ecological stress, environmental distress, climate change distress, eco-angst, climate change worry, climate anxiety, eco-anxiety, and solastalgia (Clayton et al., 2017; Coffey et al., 2021; Ojala et al., 2021). Gaps in climate anxiety research include the lack of a comprehensive theoretical framework and a single accepted definition of the term (Mah et al., 2020; Pihkala, 2020a). However, the general consensus is that climate anxiety comprises a range of distressing emotions linked to the changing environment and is non-pathological in nature, although it may impact subjective well-being (Hickman, 2020; Hickman et al., 2021; Lawrance et al., 2022). Climate anxiety is considered to be a reasonable and proportionate response to the magnitude of the climate crisis and, like any stress response, can have adaptive and productive consequences such as the adoption of more pro-environmental behaviors (Clayton, 2020; Pihkala, 2020a; Ogunbode et al., 2022). In line with the different conceptualizations, several psychometric scales have been developed to measure climate anxiety (e.g., Clayton and Karazsia, 2020; Hogg et al., 2021).

Research globally has shown that climate anxiety is particularly prevalent among young people – typically meaning adolescents and young adults (Ojala, 2012a; Clayton and Karazsia, 2020; Hickman et al., 2021; Ma et al., 2022). The anxiety they experience is understandable given they face an uncertain future, and feel betrayed and unsupported by those who hold the power to enact change (Hickman et al., 2021). The literature also suggests a high degree of engagement by young people, e.g., school strikes and climate activism (Hickman, 2020; Wallis and Loy, 2021; Bodin and Björklund, 2022; Ogunbode et al., 2022; Schwartz et al., 2022). The association between increased distress and engagement in climate change suggests that having an emotional reaction to climate change constitutes a productive and – arguably – proportional reaction to a real threat. Some of the emerging evidence suggests that climate-induced distress can be a motivator for adopting problem-solving attitudes, pro-environmental behaviors and collective action (Reser et al., 2012; Verplanken and Roy, 2013; Clayton and Karazsia, 2020; Lawrance et al., 2022; Sciberras and Fernando, 2022; Whitmarsh et al., 2022). The latter in particular may act as a buffer against poor mental health outcomes (Schwartz et al., 2022).

One area of growing interest concerns how young people cope with climate anxiety (Ojala, 2012a,b, 2013; Ojala and Bengtsson, 2019). Coping is defined as adapting cognition and behaviors to manage specific internal or external demands challenging one’s resources (Lazarus and Folkman, 1984). Coping is process-oriented and dynamic: strategies are actioned in response to a situation, vary depending on context and have secondary effects on an individual’s behavior and psychological well-being (Lazarus and Folkman, 1984). Coping efforts can be adaptive and help ameliorate harmful physical, behavioral or mental health effects of stress and adversity (Smith and Carlson, 1997; Compas et al., 2001), or coping efforts can be maladaptive and sustain or even exacerbate the negative effects of stressors.

The three main climate anxiety coping strategies are problem-focused, emotion-focused and meaning-focused coping (Ojala and Bengtsson, 2019; Russell and Victoria, 2022). Problem-focused coping involves techniques that address the problem directly, such as taking action (e.g., activism, lifestyle changes, pro-environmental behaviors) or seeking information (Lazarus and Folkman, 1984; Ojala, 2013). Emotion-focused coping involves behaviors that soothe negative emotions, including avoidance, disengagement, dismissing individual responsibility, venting emotions and outright denial (Lazarus and Folkman, 1984; Ojala, 2013; Stanisławski, 2019). Ultimately, both problem-and emotion-focused coping focus on managing and reducing distress, disregarding positive emotions (Ojala and Bengtsson, 2019). Meaning-focused coping, on the other hand, incorporates positive psychological states (Folkman, 1997, 2008), and cognitions that endorse positive emotions, including spirituality, believing that actions have an effect and expressing existential hope (Folkman and Moskowitz, 2007; Ojala, 2012a; Stanisławski, 2019). Meaning-focused coping buffers negative emotions and stimulates problem-solving (Ojala, 2012a). Existing climate anxiety literature reveals that young people may engage in all three strategies, and that there is some individual variation in the frequency with which these strategies are employed (Ojala, 2012a; Kelly, 2017; Ojala and Bengtsson, 2019; Schwartz et al., 2022).

It is anticipated that climate anxiety will continue to increase among young people globally, especially as government actions fall behind on targets (Hickman et al., 2021). However, specific vulnerabilities have yet to be examined (Lawrance et al., 2022). One particular subset of young people who may experience higher levels of climate anxiety is those choosing to pursue education and careers in the environmental sector. They are more likely to be exposed on a routine basis to potentially distressing climate data and participate in discussions on the matter. Amidst the increase in literature on climate anxiety in youth, there is a paucity of research specific to university students. While planning for the future is particularly relevant for young adults, given that major life decisions and events occur over a short period in the domains of education, family planning, career goals, health and significant purchases (Seginer, 2009; Lindstrom Johnson et al., 2014), little is known about how they are managing the emotional weight of climate change engagement in these decisions. Furthermore, although climate change has been identified as a factor in future planning (Schneider-Mayerson, 2022; Smith et al., 2022), research is scarce regarding the link between an individual’s level of climate anxiety and their consideration of climate change in future plans. Determining the extent to which young adults employ specific coping strategies and to which they incorporate climate-related considerations in their decision-making on major life events such as family planning, where to live and in the context of career, financial and travel choices, could inform tailored guidance and the provision of support in this population.

The present study aimed to assess climate anxiety, coping strategies and consideration of climate change in future plans among UK university students. We also wanted to probe whether students pursuing a degree in an environmental (or cognate) field differed on these outcome variables, compared to those in non-environmental degree programmes. This study was exploratory in nature and as such, did not test a psychological theory.

Our first research question was whether climate anxiety would be raised among environmental degree students. We hypothesized this would be the case, based on the assumption that environmental degree students are more frequently exposed to climate change news through their studies (Maran and Begotti, 2021), possess an intrinsic motivation to address environmental issues (Fraser et al., 2013) and are more connected to nature (Lankenau, 2018), all of which has been linked to increased climate anxiety.

Our second research question focused on variation in the use of coping strategies. We hypothesized that due to their active engagement with environmental issues, environmental degree students would show greater problem-focused coping, and less emotion-focused coping, as the latter promotes avoidance, distancing and denial to ward off negative feelings. Meaning-focused coping was anticipated to be elevated in environmental degree students as they may derive meaning and personal benefit from engaging with the difficult challenge of facing a climate-changed future.

Finally, we were not aware of any literature specifically examining future planning in relation to climate concerns among students. Instead of formulating specific hypotheses, we explored the effect of degree focus (environmental vs. non-environmental) and climate anxiety on decision-making around various life events.

## 2. Materials and methods

### 2.1. Data collection

Data were collected from 617 respondents between June 20 and July 21, 2022. The online questionnaire had a median response time of 7.76 min. Of the 617 responses, 71 were removed because they did not provide consent (*n* = 8); did not identify as students (*n =* 30); or were not UK-based (*n =* 33). The remaining 546 responses were manually screened to verify completion of at minimum one of the climate anxiety, coping or future planning scales. We also removed duplicate responses and respondents who straightlined (Kim et al., 2019) or sped through the survey (less than 1/3 of the median response time: 2.59 min; Zhang and Conrad, 2014). Manual screening excluded 73 respondents. In total, 473 responses were included in the analyses.

We used a purposive sampling strategy. To be eligible for the study, participants had to be a current undergraduate or postgraduate student at a UK higher education institution. We did not specify an age limit. Administrative and academic faculty members from 18 universities in the Greater London region were contacted and asked to distribute the questionnaire link among their student cohorts. Respondents were recruited via university email, departmental newsletters and institutional social media platforms (Twitter, Instagram). Additionally, eight mental health organizations were contacted. See Supplementary Table S1 for further details. Personal social media accounts were employed to widely advertise the questionnaire link via targeted messaging and publicized posts. Snowball sampling was also utilized: university faculty and members of the public were encouraged to share the questionnaire with eligible respondents.

Respondents had the opportunity to enter a prize draw for one of four £10 Grind Coffee e-gift cards. This incentive was offered to maximize the number of responses and facilitate a more accurate evaluation of the perceptions within the UK student population, particularly among those in non-environmental degree programmes who may, according to leverage-salience theory, be less intrinsically motivated to participate in climate research (Groves et al., 2004). Gift card recipients were randomly selected and notified in August 2022.

### 2.2. Survey development

The online questionnaire was implemented on the Qualtrics platform and contained a combination of validated scales and questions created for this study; for the full questionnaire, see https://osf.io/r3hpj. The questionnaire consisted of 49 questions, including multiple-choice, Likert-type and open-ended questions. The questionnaire was piloted with nine students: five who studied environmental postgraduate degrees, and four who studied non-environmental undergraduate degrees. Only minor changes to the instructions and survey wording were made.

#### 2.2.1. Demographic information

Basic demographic information was collected via multiple-choice and open-ended questions, including respondents’ gender, age, country of origin, ethnicity/cultural background, study status (full- vs. part-time), level of education being pursued, programme of study and university. Climate change beliefs were probed via multiple-choice and Likert-type questions to indicate baseline pro-environmental identity and identify potential explanatory factors, based on correlations found in previous studies (Fielding et al., 2012; Hornsey et al., 2016). The Likert-type questions employed a five-point scale ranging from *strongly disagree* to *strongly agree*.

#### 2.2.2. Climate anxiety

We used the Climate Anxiety Scale (CAS; Clayton and Karazsia, 2020) to assess the extent of climate anxiety. Respondents were asked to rate 13 statements probing cognitive and functional impairment contributed by one’s thoughts and feelings on climate change, using a five-point Likert scale. While the majority of standardized scales measuring climate or eco-anxiety focus exclusively on affective aspects (Hogg et al., 2021), the CAS scores are indicative of how day-to-day experiences are impacted by climate anxiety. The CAS is composed of two subscales representing Cognitive-Emotional Impairment (CEI) and functional impairment (FI), although the total climate anxiety score was the main focus of our analyses.

#### 2.2.3. Coping

Respondents’ coping strategies were identified using the coping scale developed by Ojala (2013) as well as one open-ended question created for this study (qualitative analyses of the open-ended question are not reported here). The coping scale included the prompt: “When one hears about societal problems such as climate change, one can feel worried or upset. Below is a list and for every item, please indicate how well it applies to what you do or think when you are reminded of climate change. Choose the alternative that you feel best applies to you, and choose only one alternative per item.” The 14-item coping scale was intended to measure the extent to which respondents employed the three major coping strategies (problem-, emotion- and meaning-focused coping) in the context of climate change. A mean item score was calculated for each coping strategy (as different numbers of items comprise the subscales).

#### 2.2.4. Future planning

The extent to which climate change is considered in future plans was identified using five Likert-type items and one open-ended question created with reference to Seginer (2009) (qualitative analyses of the open-ended question are not reported here). We asked respondents to rate how often they considered climate change when making decisions about specific domains. These include family planning, long-term habitation, career, financial and travel decisions.

### 2.3. Ethics

The confidentiality of all respondents was maintained throughout the study via the aggregation of quantitative data. A downloadable Participant Information Sheet and Consent Form were accessible, and respondents had the opportunity to ask questions. The study involved human participants and was reviewed and approved by the Centre for Environmental Policy Research Ethics Panel at Imperial College London on May 30, 2022. The participants provided their written informed consent to participate in this study.

## 3. Results

### 3.1. Sample demographics

Just over half of the sample identified as female (53.7%), and 43% identified as male, while 1.9% identified as non-binary (0.8% preferred not to disclose). The average age of the sample was 24.49 years (SD = 6.10). The majority of the sample were of European heritage/white (64%), followed by students of Asian heritage (20%) and those identifying as mixed-race or multiple heritage (8.9%). All students were currently resident in the UK, but only 37% listed it as their country of origin. The largest non-UK student group was made of Europeans (28.5%). The sample was almost evenly split between undergraduate students and master’s/postgraduate students (42.5% and 38.3%), while 18.8% were pursuing a PhD (0.4% listed “other”). Based on self-report, 249 respondents were currently studying a degree with an environmental focus (environmental group), while the remaining 224 respondents were non-environmental degree students (non-environmental group). See Supplementary Table S2 for complete demographic details of the sample.

Demographic representation was similar between the environmental and non-environmental groups. Pearson’s Chi-Square tests established that there was no significant difference between the environmental and non-environmental groups in terms of *gender* (male vs. minority groups) χ^2^(1) = 2.503, *p* = 0.114, $\varphi_{c}$ =0.073 (*n* = 469), *ethnicity* (white vs. minority groups), χ^2^(1) = 0.868, *p* = 0.351, $\varphi_{c}$ =0.043 (*n* = 472), *study status*, χ^2^(1) = 0.052, *p* = 0.820, $\varphi_{c}$ =0.011 (*n* = 473), and *level of education* (undergraduate vs. postgraduate students), χ^2^(1) = 0.117, *p* = 0.732, $\varphi_{c}$ =0.016 (*n* = 471). An independent samples t-test revealed that, on average, the environmental group (*n* = 249) was slightly older (*M* = 25.07, SD = 6.602 years) than the non-environmental group (*n* = 224) (*M* = 23.84, SD = 5.432 years). This difference was statistically significant, *t* (467.335) = −2.211, *p* = 0.028, and represented a small effect size, *d* = 0.202. We note that the “60+ years” option in the survey was replaced with 60 to compute mean ages (only three respondents fell into this category).

**Figure 1:**
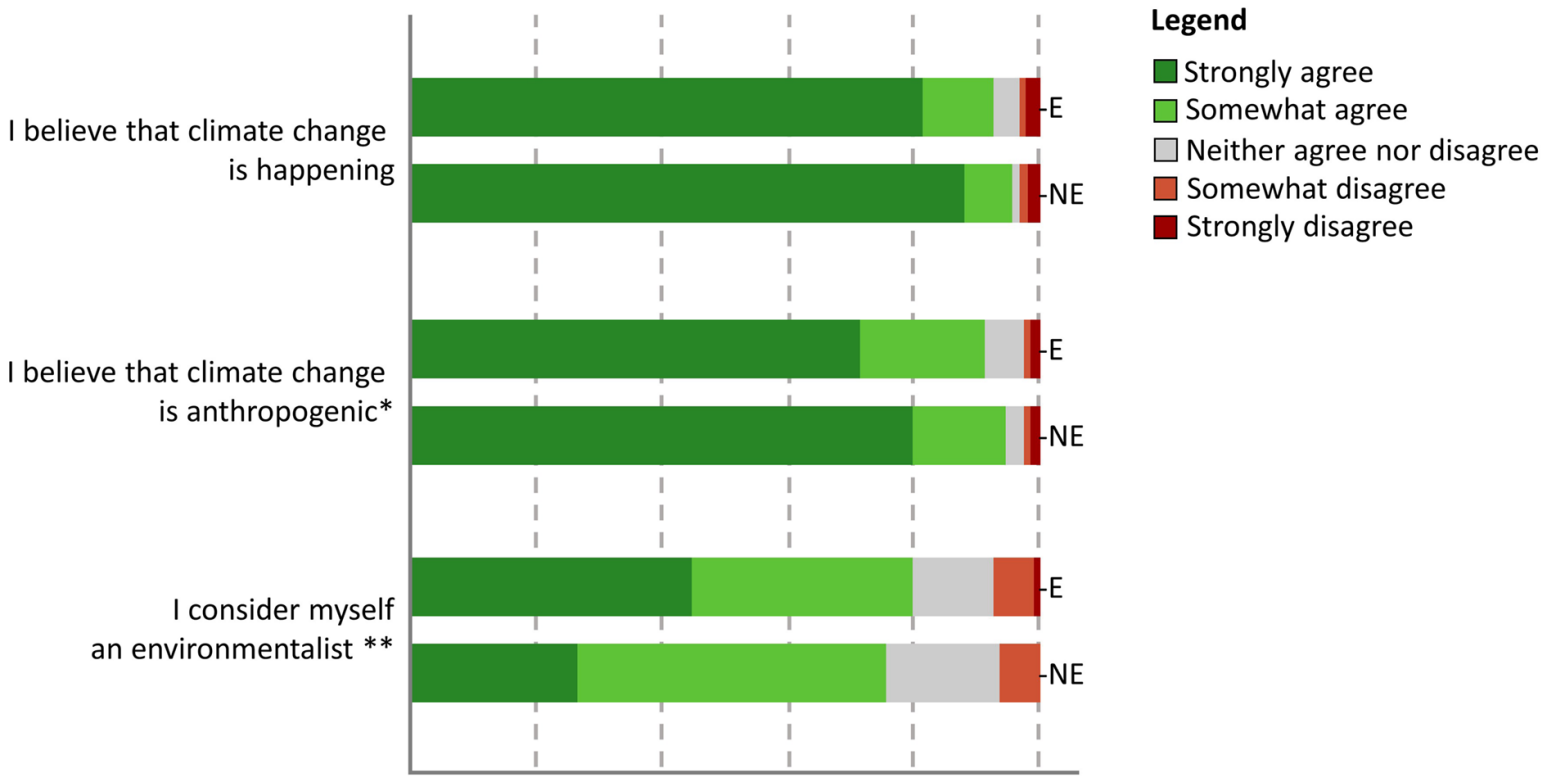
Environmental beliefs of the questionnaire sample. Non-environmental (*n* = 224) and environmental (*n* = 249) degree students were similar in their beliefs about climate change, but environmental degree students were more likely to strongly identify as “environmentalists.” **p < 0.01, *p < 0.05.

The sample indicated overall strong agreement with climate change being real (94.2% agreed or strongly agreed) and anthropogenic (92.9% agreed or strongly agreed) and a relatively large proportion self-identified as environmentalists (77.8%). Climate change beliefs were comparable between the environmental and non-environmental groups (see Figure 1). Pearson’s Chi-Square tests were used to determine whether the proportion of (dis)agreement varied between fields of study. We observed no statistically significant difference in terms of *belief that climate change is happening,* χ^2^(4) = 9.493, *p* = 0.050, $\varphi_{c}$ =0.142. Interestingly, environmental degree students seemed slightly more ambivalent about their *belief in anthropogenic climate change,* χ^2^(4) = 9.708, *p* = 0.046, $\varphi_{c}$ =0.143, but they also more strongly agreed to personal *identification as an environmentalist*, χ^2^(4) = 17.390, *p* = 0.002, $\varphi_{c}$ =0.192.

### 3.2. Outcome variables

Detailed descriptives for the outcome variables are provided in Supplementary Table S3.

#### 3.2.1. Climate anxiety

The Climate Anxiety Scale had very good internal consistency, Cronbach’s *α* = 0.925. Internal consistency for the subscales was good as well: CEI Cronbach’s *α* = 0.873, FI Cronbach’s *α* = 0.892 (for further details, see Supplementary Table S4). An independent measures t-test revealed that, on average, the environmental group (*n* = 248) was more climate anxious (*M* = 26.79, SD = 10.33) than the non-environmental group (*n* = 224) (*M* = 21.28, SD = 7.94), representing a statistically significant difference, *t* (458.539) = 6.531, *p* < 0.001, and a medium effect size, *d* = 0.594. The group differences were observed on both subscales. The environmental group scored significantly higher on the CEI subscale (*M* = 16.65, SD = 6.15) than the non-environmental group (*M* = 13.04, SD = 4.78), *t* (460.002) = 7.156, *p* < 0.001, *d* = 0.650. The environmental group also scored significantly higher on the FI subscale (*M* = 10.14, SD = 4.77) than the non-environmental group (*M* = 8.24, SD =3.83), *t* (463.677) = 4.739, *p* < 0.001, *d* = 0.436. The CAS scores for the environmental degree students were elevated compared to those reported in the development of the scale (Clayton and Karazsia, 2020), as well as recent observations from the general population of adults in Germany (Wullenkord et al., 2021), Poland (Larionow et al., 2022) and Italy (Innocenti et al., 2021), but similar to findings from young people in the Philippines (Reyes et al., 2021) and a wider sample from Europe and North Africa (Heeren et al., 2022). CAS scores for the non-environmental degree students were more similar to those previously reported.

**Figure 2:**
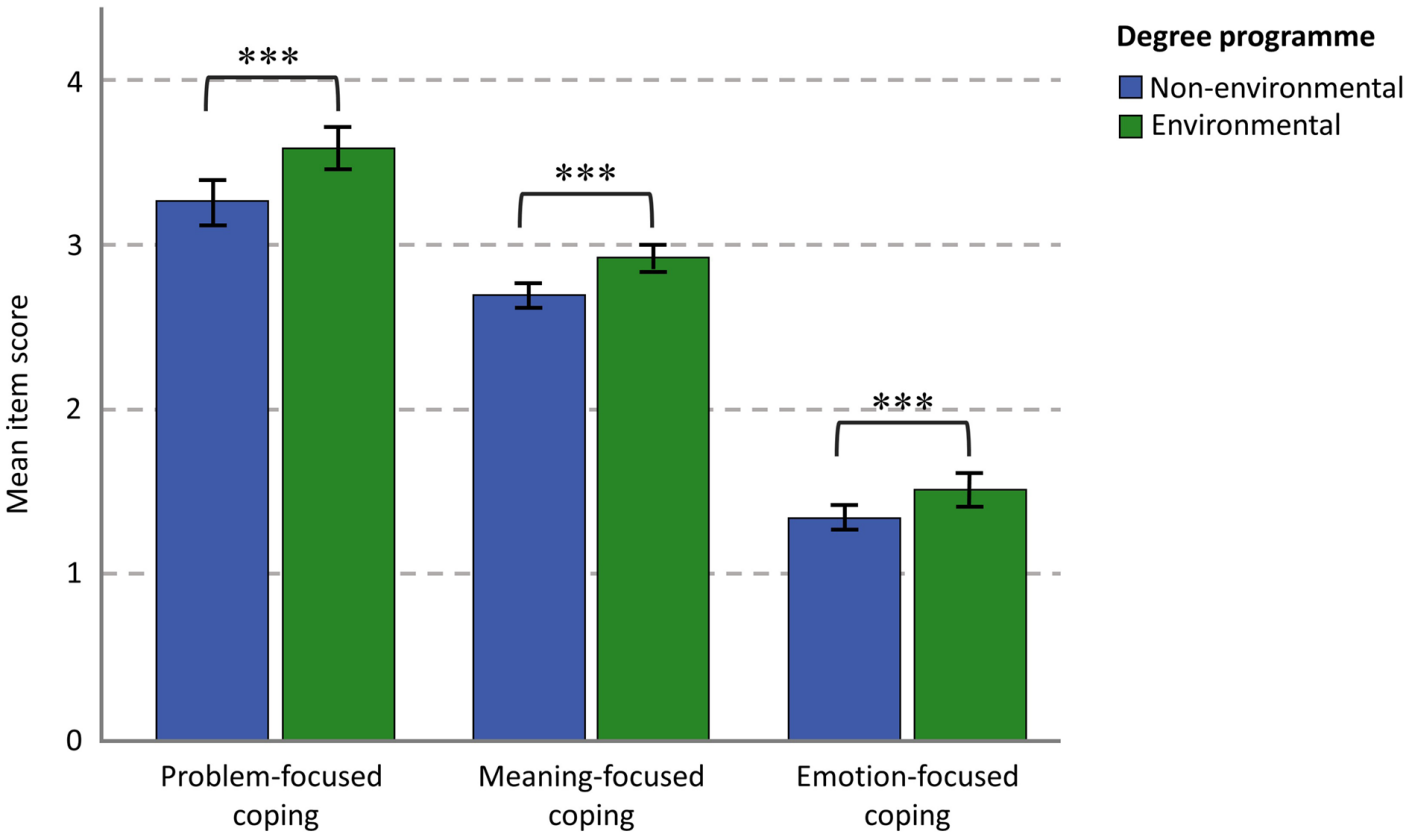
Reported endorsement of specific coping strategies by field of study. Environmental degree students (*n* = 249) reported more frequent usage of each of the three coping strategies compared to non-environmental degree students (*n* = 224). Overall, problem-focused coping was the most commonly employed strategy for dealing with one’s thoughts and feelings on climate change. ***p < 0.001.

#### 3.2.2. Coping

The coping scales had good to very good internal consistency, Cronbach’s *α* = 0.888 for the problem-focused subscale, Cronbach’s *α*  = 0.738 for the meaning-focused subscale and Cronbach’s *α* = 0.886 for the emotion-focused subscale. To explore the association with climate anxiety, we first conducted non-parametric correlation analyses between the total CAS score, as well as the CEI and FI subscale scores and the three coping scales (Table 1). There was only a moderate (for the environmental degree students) to strong (for the non-environmental degree students) correlation between climate anxiety and problem-focused coping. The other coping scales did not correlate substantively with climate anxiety.

**Table 1:** Spearman rank order correlations between the Climate Anxiety Scale (CAS) scores, its subscales, the Cognitive-Emotional Impairment (CEI) and functional impairment scores and the three coping scales.

		1	2	3	4	5
Environmental degree students (*n* = 249)	1. CAS total					
	2. CEI	0.957***				
	3. FI	0.923***	0.782***			
	4. Problem-focused coping	0.329***	0.308***	0.313***		
	5. Meaning-focused coping	0.075	0.086	0.053	0.209***	
	6. Emotion-focused coping	0.119	0.102	0.124	−0.310***	−0.008
Non-environmental degree students (*n* = 224)	1. CAS total					
	2. CEI	0.939***				
	3. FI	0.892***	0.704***			
	4. Problem-focused coping	0.545***	0.488***	0.497***		
	5. Meaning-focused coping	−0.008	−0.038	0.009	0.143*	
	6. Emotion-focused coping	−0.340***	−0.328***	−0.278***	−0.402***	0.184**

The correlations were calculated separately for the two participant groups. * < 0.05, ** < 0.01, *** < 0.001.

To understand the effect of the degree focus on coping, we conducted a 2 × 3 Mixed ANOVA (between-subjects factor: environmental vs. non-environmental field of study; within-subjects factor: problem-focused, emotion-focused, meaning-focused coping) with a Greenhouse–Geisser correction to examine differences in coping strategies between the student samples. We analyzed mean item scores to account for the fact that the subscales have different numbers of items and facilitate direct comparisons between the subscales. There was a significant effect of coping strategy, *F* (1.506, 691.172) = 801.683, *p* < 0.001, partial $\eta^{2}$ = 0.636. All Bonferroni-adjusted pairwise comparisons were significant (*p* < 0.001): problem-focused coping (*M* = 3.431, SE = 0.047) was more frequently endorsed than meaning-focused coping (*M* = 2.819, SE = 0.029) and emotion-focused coping (*M* = 1.445, SE = 0.033), and meaning-focused coping was more frequently endorsed than emotion-focused coping. There was also a significant effect of the field of study showing environmental degree students (*n* = 246) more frequently endorsed each coping strategy than non-environmental degree students (*n* = 215), *F* (1, 459) = 37.681, *p* < 0.001, partial $\eta^{2}$ = 0.076. All univariate tests comparing student groups were statistically significant, showing that, on average, environmental degree students more frequently endorsed *problem-focused coping* (*M* = 3.612, SE = 0.064) than non-environmental degree students (*M* = 3.250, SE = 0.069), *F* (1,459) = 14.886, *p* < 0.001, partial $\eta^{2}$ = 0.031; *meaning-focused coping* (*M* = 2.948, SE = 0.040) than non-environmental degree students (*M* = 2.691, SE = 0.043), *F* (1,459) = 19.262, *p* < 0.001, partial $\eta^{2}$ = 0.040, and *emotion-focused coping* (*M* = 1.559, SE = 0.045) than non-environmental degree students (*M* = 1.331, SE = 0.048), *F* (1,459) = 11.814, *p* < 0.001, partial $\eta^{2}$ = 0.025 (See Figure 2). There was no significant interaction effect between the field of study and the coping strategy, indicating that the relative frequency with which coping strategies are used was similar between environmental and non-environmental degree students, *F* (1.506, 691.172) = 0.974, *p* = 0.357, partial $\eta^{2}$ = 0.002. To explore the extent to which the between-group effects could be explained by differences in climate anxiety, we also conducted an ANCOVA, including the total CAS score as a covariate. The difference in problem-focused coping between groups was no longer significant, but meaning-focused and emotion-focused coping strategies remained significantly more endorsed by the environmental degree students (Supplementary Analysis S1 and Supplementary Figure S1).

#### 3.2.3. Decision-making about future life events

While the questions concerning decisions about future life events did not constitute an existing scale, we did conduct an internal consistency analysis to test whether the items measured a similar underlying construct. The five questions had good internal consistency, Cronbach’s *α* = 0.833 (for further details, see Supplementary Table S4).

**Figure 3:**
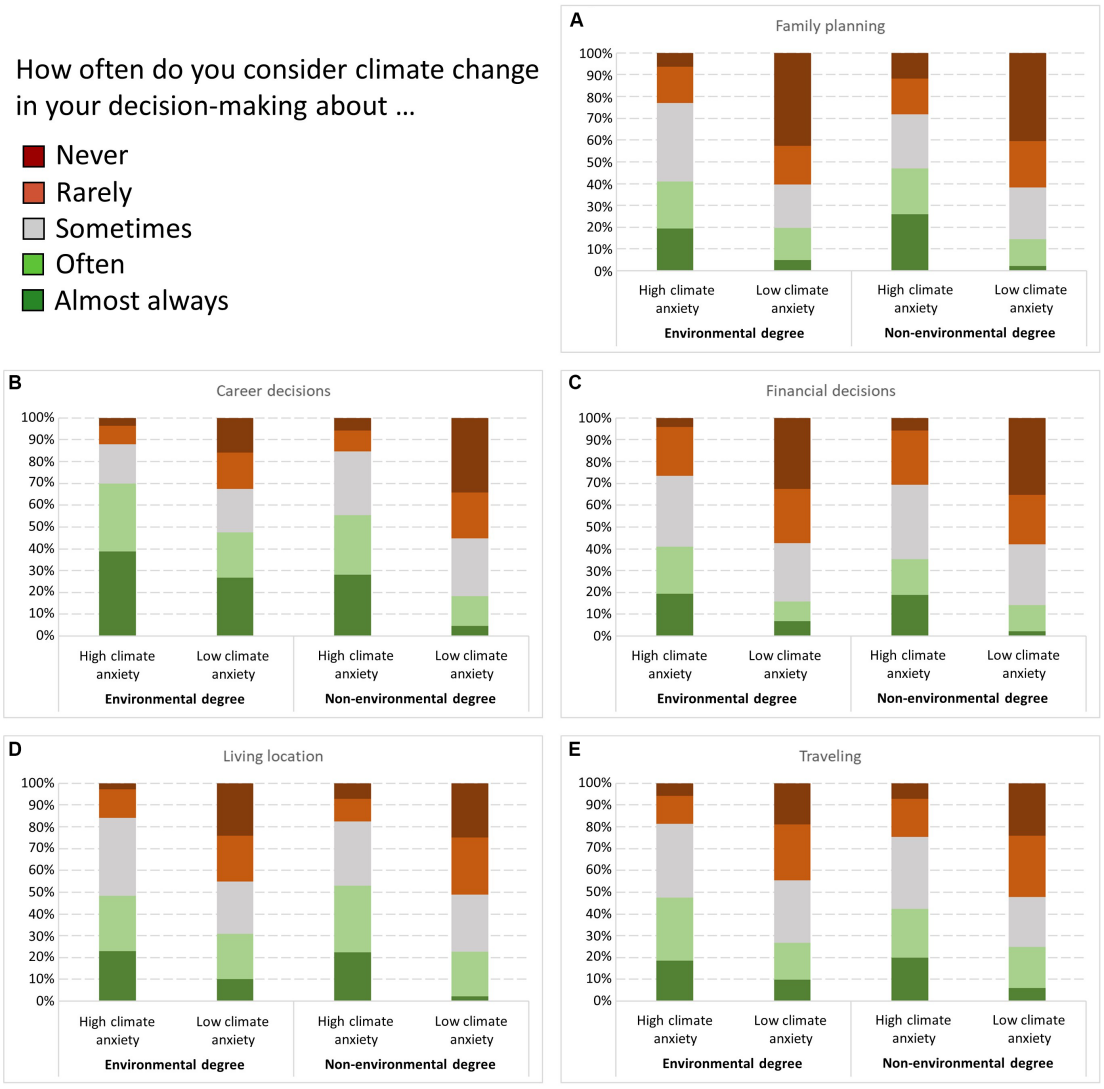
Consideration of climate change in future domains by field of study and level of climate anxiety. Percentage of environmental (*n* = 249) and non-environmental (*n* = 224) degree students experiencing high or low climate anxiety who considered climate change (never – almost always) in their decision making about five future domains: **(A)** family planning, **(B)** career decisions, **(C)** financial decisions, **(D)** living location, and **(E)** traveling. Climate anxiety was consistently associated with stronger consideration of climate change in decision-making. Field of study only affected career choices.

We conducted separate ordinal regression models on the future planning domain questions, including field of study as well as climate anxiety level as predictors. To facilitate interpretation, we transformed the climate anxiety variable from a continuous to a binary variable using a median split. There was no evidence of multicollinearity between predictor variables (VIF < 2). For each regression model, we also assessed the proportional odds assumption. Figure 3 shows the descriptive data for the comparison.

For family planning, the model was significantly better than the null model, χ^2^(2) = 85.409, *p* < 0.001, and achieved a good fit, Pearson χ^2^(10) = 15.643, *p* = 0.110. The proportional odds assumption was met, χ^2^(6) = 9.433, *p* = 0.151. Overall, the predictors explained 16.4% of the variance (Nagelkerke *R*^2^). The cumulative odds of highly climate anxious respondents were 4.686 (95% CI, 3.262–6.731) times that of low-anxious respondents, Wald χ^2^(1) = 69.845, *p* < 0.001. However, there was no significant difference between fields of study, Wald χ^2^(1) = 0.154, *p* = 0.695.

For decisions about where to live, the model was significantly better than the null model, χ^2^(2) = 59.330, *p* < 0.001, achieving an acceptable fit, Pearson χ^2^(10) = 18.085, *p* = 0.054. The proportional odds assumption was met, χ^2^(6) = 11.026, *p* = 0.088. Overall, the predictors explained 12.8% of the variance (Nagelkerke *R*^2^). The cumulative odds of highly climate anxious respondents were 3.671 (95% CI, 2.574–5.237) times that of low-anxious respondents, Wald χ^2^(1) = 51.486, *p* < 0.001. However, there was no significant difference between fields of study, Wald χ^2^(1) = 0.329, *p* = 0.566.

For career decisions, the model was significantly better than the null model, χ^2^(2) = 87.655, *p* < 0.001, achieving a good fit, Pearson χ^2^(10) = 15.524, *p* = 0.114. The proportional odds assumption was met, χ^2^(6) = 5.958, *p* = 0.428. Overall, the predictors explained 18.3% of the variance (Nagelkerke *R*^2^) and both predictors had a significant effect on the outcome variable. The cumulative odds of highly climate anxious respondents were 3.472 (95% CI, 2.441–4.939) times that of low-anxious respondents, Wald χ^2^(1) = 47.937, *p* < 0.001. The cumulative odds of environmental degree students were 2.296 (95% CI, 1.628–3.239) times that of non-environmental degree students, Wald χ^2^(1) = 26.645, *p* < 0.001.

For financial decisions, the model was significantly better than the null model, χ^2^(2) = 58.687, *p* < 0.001, and achieved an acceptable fit, Pearson χ^2^(10) = 16.626, *p* = 0.083. The test of proportional odds further indicated some caution is warranted regarding the appropriateness of the model, χ^2^(6) = 12.853, *p* = 0.045. Overall, the predictors explained 12.7% of the variance (Nagelkerke *R*^2^). The cumulative odds of highly climate anxious respondents were 3.663 (95% CI, 2.575–5.211) times that of low-anxious respondents, Wald χ^2^(1) =52.157, *p* < 0.001. However, there was no significant difference between fields of study, Wald χ^2^(1) = 0.230, *p* = 0.631.

For traveling, the model was significantly better than the null model, χ^2^(2) = 39.673, *p* < 0.001, and achieved a good fit, Pearson χ^2^(10) = 6.280, *p* = 0.799. The proportional odds assumption was met, χ^2^(6) = 4.647, *p* = 0.590. Overall, the predictors explained 8.7% of the variance (Nagelkerke *R*^2^). The cumulative odds of highly climate anxious respondents were 2.755 (95% CI, 1.949–3.894) times that of low-anxious respondents, Wald χ^2^(1) = 32.948, *p* < 0.001. However, there was no significant difference between fields of study, Wald χ^2^(1) = 1.415, *p* = 0.234.

## 4. Discussion

Climate anxiety is expected to increase in prevalence and certain populations, including young people, individuals who feel most closely connected to nature and those who work in environmental protection or related careers, remain disproportionately burdened (Fraser et al., 2013; Clayton, 2018; Coffey et al., 2021; Hickman et al., 2021). In this study, we aimed to understand the occurrence and individual management of climate anxiety in UK university students. Specifically, we explored whether distress, coping strategies and consideration of climate change in future plans differed depending on the field of study. Environmental degree students reported higher levels of climate anxiety, greater endorsement of all three coping strategies and greater consideration of climate change in career plans compared to non-environmental degree students. The sample most frequently employed problem-focused coping. Regardless of their degree focus, highly climate-anxious students were more likely to consider climate change in their future plans compared to students with low levels of climate anxiety. To our knowledge, this is the first study to investigate the association between climate anxiety, coping strategies and future planning in university students.

### 4.1. Impact of the field of study on climate anxiety

In agreement with our initial hypothesis, environmental degree students reported higher levels of climate anxiety than non-environmental degree students. This is analogous to findings from a previous study of Australian university students, in which environmental degree students reported higher levels of stress related to climate change and the state of the world than non-environmental degree students (Kelly, 2017). The potential rationale for climate anxiety in environmental professionals includes more frequent exposure to climate change evidence, an increased connection to the natural world and an intrinsic motivation to address environmental issues (Fraser et al., 2013; Lankenau, 2018; Clayton and Karazsia, 2020; Maran and Begotti, 2021). Similarly, environmental courses are likely exposing students to more information about climate change threats and this may act as a chronic, indirect impact of climate change (Hrabok et al., 2020). Pursuance of an environmentally focused education arguably indicates engagement with environmental affairs and personal interest in climate issues. While the directionality of this effect remains under debate to some extent, it is unsurprising that environmental degree students are more climate anxious than their non-environmental counterparts.

The overall Climate Anxiety Scale (CAS) score and mean CEI and FI item scores of the environmental degree students were generally higher than those reported among adult European samples in the literature (Innocenti et al., 2021; Larionow et al., 2022). It is worth acknowledging that it is difficult to compare CAS scores across studies, in part because the underlying measurement model remains somewhat debated (Hogg et al., 2023). More validation studies are required to better understand the stability of climate anxiety scores, what constitutes an “elevated” score and whether certain interest groups, such as students of environmental science, consistently demonstrated higher climate anxiety compared to other (young) adults.

Though climate anxiety often elicits a range of “negative” or distressing emotions, the literature generally concurs that this response is proportional to the scale and uncertain nature of the threat of climate change (Clayton, 2020; Clayton and Karazsia, 2020). In this study, we provide a snapshot of university students before they enter their careers. We are uncertain whether cumulative exposure and engagement with environmental issues have an effect on people’s well-being because currently, there is no longitudinal research in this area. Further investigation is therefore warranted, such as via follow-up studies involving interviews to further inform climate anxiety prevalence over time, control for responses to short-term environmental events (Caruana et al., 2015) and contribute valuable insight into which coping strategies bolster mental well-being over the long term.

These findings are relevant to university students. Recognizing that other students are climate anxious can de-stigmatize climate anxiety and help climate-anxious students feel supported. Additionally, increased agency and climate action may result when students learn to assess their coping strategies and incorporate productive adaptive strategies. The current study indicates that environmental degree students are especially vulnerable, suggesting this group would benefit from increased support in the form of information sharing, discussion and coordinated collective activities (Russell and Victoria, 2022). Prioritizing the mental well-being of university students will strengthen their capacity to enact impactful, wide-scale change.

### 4.2. Coping with climate anxiety

The most commonly employed coping strategy was problem-focused coping, followed by meaning-focused, while the avoidance tactics of emotion-focused coping were relatively uncommon. We also observed associations between specific coping strategies and the “severity” of climate anxiety, with some notable differences between the environmental and non-environmental degree students. The cross-sectional design does not allow us to draw conclusions about the direction of effect, but it does point to potential explanations that are deserving of further research attention. Problem-focused coping was positively, and moderately to strongly associated with climate anxiety. This finding is expected given the multiple benefits of taking climate action and is in line with previous climate anxiety studies (Kelly, 2017; Ojala and Bengtsson, 2019; Schwartz et al., 2022). The sample of young people we studied thus seem to be turning to adaptive and constructive responses to manage increasing distress levels. There are a number of factors that may contribute to this. For example, positive, solution-oriented communication, such as that which is employed in an academic context and amongst post-secondary peers, is correlated with problem-focused coping styles in young people (Seko and Lau, 2022). However, in one study, students who perceived an insufficient provision of solutions turned to problem-focused strategies to enact their own (Kelly, 2017). This is consistent with the particularly strong link between climate anxiety and problem-focused coping among the non-environmental degree students. This group, whose day-to-day does not revolve around solving environmental problems, nevertheless appears to turn to action when climate anxiety is roused.

Perhaps unsurprisingly, emotion-focused coping was negatively linked to climate anxiety, but only in non-environmental degree students. Whether distraction and avoidance reduce anxiety, or lower levels of concern among this group may lead to denialist attitudes is unclear. Although environmental degree students were more likely than their non-environmental counterparts to report using emotion-focused coping, it was not linked to higher (or lower) climate anxiety. This coping strategy therefore seems to be a function of the context in which this group operates, rather than fundamentally linked to their distress around climate change. While this is a speculative interpretation, the timing of the study, occurring as the COVID-19 pandemic was coming to an end, may have contributed to young people having little remaining emotional resilience, encouraging them to rely in part on avoidance and minimization. For those studying environmental degrees, the inability to “escape” from exposure to environmental doom in their everyday (e.g., through course content, interactions with others), and their increased awareness of the sheer scale of the problem, may have presented an additional challenge on their alternative coping resources such as those focused on enacting solutions.

The observation that the environmental degree students reported more frequent use of coping strategies in general suggests that they are actively managing their thoughts and feelings around climate change. Even when controlling for increased rates of climate anxiety in this group, the environmental degree students reported more frequent use of coping strategies, particularly through meaning-and emotion-focused means. Whether they are more intrinsically motivated to derive sense and purpose from rising to the challenge of climate change or driven to distraction due to the unavoidable exposure to environmental messaging in their day-to-day lives, what is clear is that they are conscious of the psychological impacts. The observation that despite having multiple coping strategies at their disposal, environmental degree students on average still reported greater climate anxiety, could be taken as ineffective coping. However, coping skills may support well-being without necessarily reducing climate-specific anxiety and thus be an adaptive response. Nevertheless, the effects on longer-term well-being require more research. Problem-focused coping strategies, for instance, are useful in the short term, but may negatively impact well-being because an individual cannot solve climate change (Ojala, 2012b; Clayton, 2020; Soutar and Wand, 2022). This may place students at risk when they already feel stressed, overwhelmed and vulnerable, factors that themselves contribute to climate anxiety (Pihkala, 2020a). Emotion-focused coping may therefore be beneficial when the challenge is beyond an individual’s control as it provides well-being protection through distraction and minimization (Folkman, 2008). Meaning-focused coping, which encourages the individual to draw upon their beliefs, values and existential goals, can be an effective tool in the context of intractable problems such as climate change (Ojala, 2013). Furthermore, the knowledge and lived experience that climate anxiety can be effectively managed may bolster mental well-being and encourage students to request the support they need from their university, provided the university has relevant policies in place (Pihkala, 2020b).

We caution, that as we did not include a measure of subjective well-being, we cannot determine whether the two student groups experienced differential mental health outcomes in association with climate anxiety and different coping strategies. As far as we are aware, the use of coping strategies has not previously been contrasted between environmental students or professionals and their counterparts working in different fields. Qualitative research approaches are needed to garner deeper insights into when and why people employ certain coping strategies, and the link between coping and well-being outcomes as well as behavioral outcomes (e.g., support for environmental causes, activism).

These findings have implications not just for students, but environmental practitioners all around, who should incorporate the benefits of employing multiple coping strategies. A focus on taking action (problem-focused coping), fostering resilience (meaning-focused coping), and seeking social support in group settings away from distressing climate information (emotion-focused coping) can enhance well-being when experiencing climate anxiety (Russell and Victoria, 2022). A multi-pronged approach implicating connections with others is recommended, as it is proven to support individuals with climate anxiety (Baudon and Jachens, 2021), including environmental specialists (Clayton, 2018). Further research should focus on capacity development, particularly in the populations most vulnerable to climate anxiety. Research should also emphasize education on effective coping styles. University educators and curriculum developers, particularly those leading environmental programmes, should be advised that certain students may be at elevated risk for climate anxiety. University policies and services targeting student and staff mental health and well-being should be updated to reflect this risk and provide support. Creating professional development opportunities that facilitate the integration of climate change information into coursework across all fields of study, support the recognition of climate anxiety and increase awareness of resources to enhance coping would be advantageous (Ojala, 2016; Thew et al., 2021). Education programmes should aim to develop student’s competencies holistically. Apart from targeting students’ cognitive competences around problem understanding, definition and analysis, there is a need for coping with negative emotions, feelings of distress and anxiety, and thus affective and behavioral competences of developing resilience and taking action should be prioritized (Thew et al., 2021). In terms of curricular guidelines, learning outcomes influence decisions on why, how and what is being taught and thus targeting the health and well-being of students in environmental and sustainability programmes as one of the intended outcomes should be key (Kioupi and Voulvoulis, 2020). In addition, offering learning activities that provide university students with real-world opportunities for taking action, collaborating for solutions and reflecting on coping with emotions does not only develop skilled environmental professionals, but also resilient and empowered citizens (Kioupi and Voulvoulis, 2022). Empowering university educators to clarify their own emotions and develop their coping strategies is beneficial as academics also experience climate anxiety (Fraser et al., 2013). Supporting these leaders will promote resilience and facilitate the knowledge transfer vital to addressing the climate crisis (Thew et al., 2021).

### 4.3. Climate anxiety and the consideration of climate change in future plans

Future planning was found to be strongly affected by climate anxiety. Highly climate-anxious individuals were more likely to incorporate climate change considerations in all five domains assessed. However, career was the only domain where environmental degree students were more likely than non-environmental degree students to consider climate change. These findings demonstrate that climate anxiety is not simply an in-the-moment response to current events, but is influencing the decision-making of young people in important ways.

The implication of climate change in future plans by highly climate-anxious students may reflect their worry and perceived environmental efficacy. This agrees with general anxiety theories: anxious emotions increase attention to a threat through information seeking and discussions with others, resulting in a heightened focus on the issue and its potential consequences (Ojala et al., 2021). Thus, fixated attention on climate change may amplify its consideration in future plans. Empirical data on how climate anxiety relates to future planning is scarce. These results are novel and represent an opportunity for additional investigation. Studies that explore the interactions between climate anxiety, environmental efficacy and environmental agency and determine their role in future planning are required.

Environmental degree students were more likely than non-environmental peers to consider climate change in future career decisions. This was the only domain that significantly differed between the two groups. This finding can be attributed to feelings of personal responsibility to address the climate crisis, stemming from an increased awareness of climate change and its effects (Gallagher and Cattelino, 2020). Environmental students are pursuing education that arguably aligns with their concern for the planet; therefore, it is logical that they endeavor to apply this knowledge in their careers. This sense of personal obligation has been reported in the domain of family planning, e.g., choosing not to have children based on concerns about children’s carbon footprint and well-being in a changing world (Schneider-Mayerson and Leong, 2020). However, we did not find that environmental degree students’ concerns about climate change more strongly affected other life choices such as family planning, long-term habitation, financial decisions and travel plans. Arguably, these domains are less directly linked to the respondents’ chosen educational pathway, and decisions are likely affected by a wider range of personal circumstance, lifestyle and other social considerations.

Previous work that probed future planning in climate-conscious individuals or the general population is limited to family planning (Clayton, 2020; Schneider-Mayerson and Leong, 2020; Schneider-Mayerson, 2022). Therefore, the concept of personal responsibility remains to be verified, suggesting further research is required to identify what motivates students to consider climate change in their future plans. This study was the first to specifically assess young people’s future decision-making in the context of climate anxiety, thus more work is required to examine how these decision processes evolve over time and whether people follow through on these hypothetical future plans.

### 4.4. Limitations

The primary limitation involves the sampling approach. While our sample size was similar to other climate anxiety studies (e.g., Ojala, 2012a; Kelly, 2017; Lawrance et al., 2022), it is possible that the study was underpowered to detect small effects. The sample also represented only a small fraction of the UK post-secondary student population (which exceeds two million students) and the institutions contacted comprise a minority of the UK university network (HESA, 2022). Respondents were also overrepresented by British individuals from white ethnic groups studying at three institutions. A pragmatic approach to snowball sampling was employed as a result of the time constraint of the study; thus, institutional representation was biased by the demographics of universities in the Greater London region. Therefore, the results cannot easily be generalized to all university students. Nevertheless, this study provides a methodology that can be readily employed in other regions to widen the geographical scope of the results. Thus, the distribution of this questionnaire to a larger, more geographically diverse sample is recommended to validate the findings. Analyses involving university students in low-income countries are a priority, as few such studies exist (Charlson et al., 2021). Given the increasing young demographic in low-income regions and the unique climate impacts they face (United Nations, 2019), a deeper understanding of the prevalence of climate anxiety in this population is imperative.

This study relied on self-reporting. In environmental psychology, there is a significant variance between self-reported and objective behaviors, with objective measurements generally being less biased (Kormos and Gifford, 2014). In addition, Likert-type questions are subjective and can be interpreted differently by individuals. Respondents only completed the questionnaire once. Thus, their responses refer to a single point in time and may reflect emotions triggered by external events occurring at the time [e.g., the July 2022 heat wave (Kendon, 2022), the COVID-19 pandemic]. The strictly quantitative approach presents a limitation when exploring coping responses, as a single question does not allow for the in-depth exploration of respondents’ experiences. Follow-up interviews would contextualize the questionnaire responses and potentially lead to clarification on the complexity of the relationships between the studied outcomes, as well as insights into how beliefs and experiences with climate change shape attitudes and decisions.

One surprising finding that might warrant further investigation was the observation that beliefs about the occurrence and causation of climate change in environmental degree students were slightly less extreme. Although we noted overwhelming support for the statements “climate change is happening” and “climate change is anthropogenic,” slightly lower proportions of environmental degree students “strongly” agreed. There are several possible explanations. The first is that environmental degree students, having more in-depth knowledge, may hold a more nuanced and cautious view of the causes of climate change and their own knowledge on it. Of course, environmental degrees vary widely, and students may be more acutely aware of their relative lack of expertise in climate science specifically, leading them to underestimate or deny knowledge on the topic (as was evident in the fact that they were as likely if not more likely to display denialist and avoidant attitudes in the emotion-focused coping scale). Another plausible factor worth considering is that not all students pursuing an environmental degree have strong pro-environmental values or consider themselves environmentalists. While many do identify as such, perhaps those that do not can swing toward reactionary skepticism, resulting in more extreme attitudes among this sub-sample. It has to be acknowledged that the grouping into environmental and non-environmental degree students was a crude approach, based on self-determination. While some interesting patterns have emerged, it is also clear that the environmental field of study is diverse. Some students may not be very engaged with climate change, others may show avoidant coping patterns. Regardless, further research is needed to understand how individual interests and different motivation to study an environmental degree might shape cognitions about climate change and how these translate into life decisions, personal choices and actions.

## 5. Conclusion

Globally, youth are very concerned about climate change (Hickman et al., 2021; Lawrance et al., 2022) and identify it as the most critical social issue today (Ojala, 2018). Young people and environmental professionals report some of the highest rates of climate anxiety (Fraser et al., 2013; Clayton, 2018; Coffey et al., 2021). At the same time, research has suggested that this may be a force for good, as it motivates people to take action and engage (Ojala et al., 2021). As one of the first studies to investigate climate anxiety, coping strategies and decision-making about the future in university students pursuing environmental degrees, this study sheds light on the psychological burden carried by emerging environmental professionals. We found that compared to those studying other topics, environmental degree students have higher rates of climate anxiety, reported more frequent use of coping strategies and are actively considering career paths with climate change in mind. It was notable that the use of problem-focused coping strategies was associated with higher rates of climate anxiety, suggesting that university students in the UK are actively engaged in the issue, despite the relatively limited direct experience with climate change impact compared to other, more physically and socially vulnerable regions. Yet it remains to be determined whether this may come at the cost of maintaining subjective well-being, when climate solutions are not enacted by those with the power to make change. Climate anxious students considered climate change in all domains of future planning – family planning, long-term habitation, career, and financial and travel decisions. These findings indicate a need to monitor anxiety and cope with the challenges of the climate crisis head-on. Some young people are doing so through their study and career decisions, and their active engagement in the climate and/or environmental movements (e.g., Fridays for Future). Young people are critical stakeholders in realizing fair and equitable solutions to the climate crisis. Therefore, supporting young people’s mental well-being by empowering them to cope effectively with climate anxiety will strengthen their capacity to initiate impactful change. Emerging professionals in the environmental field may be in particular need of supportive structures and skill development; the implications for teaching and curriculum design in higher education are deserving of further attention.

## Data availability statement

Original datasets are available in a publicly accessible repository: https://osf.io/qcxrz. The original contributions presented in the study are included in the article/Supplementary material, further inquiries can be directed to the corresponding author.

## Ethics statement

The study involved human participants and was reviewed and approved by the Centre for Environmental Policy Research Ethics Panel at Imperial College London. The participants provided their written informed consent to participate in this study.

## Author contributions

CD: conceptualization, methodology, investigation, formal analysis, data curation, writing – original draft, writing – review and editing, and visualization. VK: conceptualization, methodology, writing – review and editing, supervision, and project administration. AV: conceptualization, methodology, formal analysis, data curation, writing – review and editing, visualization, supervision, and project administration. All authors contributed to the article and approved the submitted version.

## Conflict of interest

The authors declare that the research was conducted in the absence of any commercial or financial relationships that could be construed as a potential conflict of interest.

## Publisher’s note

All claims expressed in this article are solely those of the authors and do not necessarily represent those of their affiliated organizations, or those of the publisher, the editors and the reviewers. Any product that may be evaluated in this article, or claim that may be made by its manufacturer, is not guaranteed or endorsed by the publisher.
