# Supplementary material for: Climate anxiety, coping strategies and planning for the future in environmental degree students in the UK
Source: Front Psychol. 2023 Jul 26;14:1126031. doi: 10.3389/fpsyg.2023.1126031 (PMC10409990; doi:10.3389/fpsyg.2023.1126031)

Supplementary Material

Climate anxiety, coping strategies and planning for the future in environmental degree students in the UK

Cami Daeninck*, Vasiliki Kioupi, Ans Vercammen

*** Correspondence:** Cami Daeninck: cami.daeninck21@alumni.imperial.ac.uk

Supplementary Table S1. Summary of academic institutions, mental health organizations and student groups contacted to request support in distributing the online questionnaire.

Organization	Faculty	Department and Centre	Agreed to distribute?
Imperial College London	Engineering	Aeronautics	Yes
Imperial College London	Engineering	Bioengineering	Yes
Imperial College London	Engineering	Chemical Engineering	Yes
Imperial College London	Engineering	Civil & Environmental Engineering	Yes
Imperial College London	Engineering	Computing	No response
Imperial College London	Engineering	Dyson School of Design Engineering	No
Imperial College London	Engineering	Earth Science & Engineering	Yes
Imperial College London	Engineering	Electrical & Electronic Engineering	Yes
Imperial College London	Engineering	Materials	No
Imperial College London	Engineering	Mechanical Engineering	Yes
Imperial College London	Medicine	Brain Sciences	No response
Imperial College London	Medicine	Immunology & Inflammation	Yes
Imperial College London	Medicine	Infectious Disease	No response

Imperial College London	Medicine	Molecular Bacteriology & Infection	Yes
Imperial College London	Medicine	Metabolism, Digestion & Reproduction	Yes
Imperial College London	Medicine	National Heart & Lung Institute	No
Imperial College London	Medicine	School of Public Health	No response
Imperial College London	Medicine	Surgery & Cancer	No response
Imperial College London	Natural Sciences	Chemistry	Yes
Imperial College London	Natural Sciences	Mathematics	No response
Imperial College London	Natural Sciences	Physics	Yes
Imperial College London	Natural Sciences	Life Sciences	Yes
Imperial College London	Natural Sciences	Centre for Environmental Policy	Yes
Imperial College London	Business School	Finance	No
Imperial College London	Business School	Management & Entrepreneurship	No response
Imperial College London	Business School	Economics & Public Policy	No
Imperial College London	Business School	Marketing, Analytics & Operations	No response
Imperial College London		Grantham Institute – Climate Change & the Environment	Yes
University College London	Arts & Humanities		No
University College London	Bartlett (Built Environment)		No response
University College London	Brain Sciences		No response
University College London	Institute of Education		Yes
University College London	Engineering Sciences		No response

University College London	Laws		Yes
University College London	Life Sciences		No response
University College London	Mathematical & Physical Sciences		No response
University College London	Medical Sciences		No response
University College London	Population Health Sciences		No response
University College London	Social & Historical Sciences		No
King’s College London	Arts & Humanities		No response
King’s College London	Business School		No response
King’s College London	Dentistry, Oral & Craniofacial Sciences		No response
King’s College London	The Dickson Poon School of Law		No
King’s College London	Life Sciences & Medicine		No response
King’s College London	Natural, Mathematical & Engineering Sciences	Informatics	No
King’s College London	Florence Nightingale Faculty of Nursing, Midwifery & Palliative Care		No response
King’s College London	Institute of Psychiatry, Psychology & Neuroscience		No
King’s College London	Social Science & Public Policy	School of Global Affairs	No

The London School of Economics and Political Science	Accounting	Yes
The London School of Economics and Political Science	Anthropology	Yes
The London School of Economics and Political Science	Data Science Institute	Yes
The London School of Economics and Political Science	Economics	Yes
The London School of Economics and Political Science	Economic History	No response
The London School of Economics and Political Science	European Institute	No response
The London School of Economics and Political Science	Finance	No response
The London School of Economics and Political Science	Firoz Lalji Institute for Africa	No response
The London School of Economics and Political Science	Gender Studies	Yes
The London School of Economics and Political Science	Geography & Environment	Yes
The London School of Economics and Political Science	Government	No response
The London School of Economics and Political Science	Health Policy	Yes

The London School of Economics and Political Science	International Development	No
The London School of Economics and Political Science	International History	Yes
The London School of Economics and Political Science	International Inequalities Institute	No
The London School of Economics and Political Science	International Relations	Yes
The London School of Economics and Political Science	Language Centre	No
The London School of Economics and Political Science	LSE Law School	No response
The London School of Economics and Political Science	Management	No response
The London School of Economics and Political Science	Mathematics	Yes
The London School of Economics and Political Science	Media & Communications	No response
The London School of Economics and Political Science	Methodology	No response
The London School of Economics and Political Science	Philosophy, Logic & Scientific Method	No
The London School of Economics and Political Science	Psychological & Behavioural Science	No response

The London School of Economics and Political Science		School of Public Policy	No response
The London School of Economics and Political Science		Social Policy	No
The London School of Economics and Political Science		Sociology	No
The London School of Economics and Political Science		Statistics	No response
Queen Mary University of London	Humanities & Social Sciences	School of Business & Management	No response
Queen Mary University of London	Humanities & Social Sciences	School of Economics & Finance	No response
Queen Mary University of London	Humanities & Social Sciences	School of English & Drama	Yes
Queen Mary University of London	Humanities & Social Sciences	School of Languages, Linguistics & Film	No response
Queen Mary University of London	Humanities & Social Sciences	School of Geography	No
Queen Mary University of London	Humanities & Social Sciences	School of History	No
Queen Mary University of London	Humanities & Social Sciences	School of Law	No response
Queen Mary University of London	Humanities & Social Sciences	School of Politics & International Relations	No response
Queen Mary University of London	Medicine & Dentistry	The Blizard Institute, Centre for Cell Biology & Cutaneous Research	No
Queen Mary University of London	Medicine & Dentistry	The Blizard Institute, Centre for Genomics & Child Health	No response
Queen Mary University of London	Medicine & Dentistry	The Blizard Institute, Centre for Immunobiology	No response

Queen Mary University of London	Medicine & Dentistry	The Blizard Institute, Centre for Neuroscience, Surgery & Trauma	Yes
Queen Mary University of London	Medicine & Dentistry	Institute of Dentistry, Centre of Oral Bioengineering	No response
Queen Mary University of London	Medicine & Dentistry	Institute of Dentistry, Centre for Dental Public Health & Primary Care	No response
Queen Mary University of London	Medicine & Dentistry	Institute of Dentistry, Centre for Oral Clinical Research	No
Queen Mary University of London	Medicine & Dentistry	Institute of Dentistry, Centre for Oral Immunobiology & Regenerative Medicine	No
Queen Mary University of London	Medicine & Dentistry	Institute of Health Sciences Education	No
Queen Mary University of London	Medicine & Dentistry	William Harvey Research Institute, Centre for Advanced Cardiovascular Imaging	Yes
Queen Mary University of London	Medicine & Dentistry	William Harvey Research Institute, Centre for Biochemical Pharmacology	Yes
Queen Mary University of London	Medicine & Dentistry	William Harvey Research Institute, Centre for Cardiovascular Medicine & Devices	No response
Queen Mary University of London	Medicine & Dentistry	William Harvey Research Institute, Centre for Clinical Pharmacology	No
Queen Mary University of London	Medicine & Dentistry	William Harvey Research Institute, Centre for Microvascular Research	Yes
Queen Mary University of London	Medicine & Dentistry	William Harvey Research Institute, Centre for Translational Medicine & Therapeutics	No response

Queen Mary University of London	Medicine & Dentistry	William Harvey Research Institute, Centre for Sports & Exercise Medicine	No response
Queen Mary University of London	Medicine & Dentistry	Wolfson Institute of Population Health, Centre for Public Health & Policy	No response
Queen Mary University of London	Medicine & Dentistry	Wolfson Institute of Population Health, Centre for Primary Care	No
Queen Mary University of London	Medicine & Dentistry	Wolfson Institute of Population Health, Centre for Prevention, Detection & Diagnosis	Yes
Queen Mary University of London	Medicine & Dentistry	Wolfson Institute of Population Health, Centre for Psychiatry & Mental Health	Yes
Queen Mary University of London	Medicine & Dentistry	Wolfson Institute of Population Health, Centre for Evaluation & Methods	No response
Queen Mary University of London	Science & Engineering	School of Biological & Behavioural Sciences	No
Queen Mary University of London	Science & Engineering	School of Electronic Engineering & Computer Science	No
Queen Mary University of London	Science & Engineering	School of Engineering & Materials Science	No response
Queen Mary University of London	Science & Engineering	School of Mathematical Sciences	No
Queen Mary University of London	Science & Engineering	School of Physical & Chemical Sciences	No
Birkbeck, University of London	School of Arts	English, Theatre & Creative Writing	No response
Birkbeck, University of London	School of Arts	Film, Media & Cultural Studies	No response
Birkbeck, University of London	School of Arts	History of Art	No response

Birkbeck, University of London	School of Arts	Languages, Cultures & Applied Linguistics	No response
Birkbeck, University of London	School of Business, Economics & Statistics	Computer Sciences & Informatics	Yes
Birkbeck, University of London	School of Business, Economics & Statistics	Economics, Mathematics & Statistics	No response
Birkbeck, University of London	School of Business, Economics & Statistics	Management	No response
Birkbeck, University of London	School of Business, Economics & Statistics	Organizational Psychology	No response
Birkbeck, University of London	School of Law	Criminology	Yes
Birkbeck, University of London	School of Law	Law	No
Birkbeck, University of London	School of Science	Biological Sciences	No response
Birkbeck, University of London	School of Science	Earth & Planetary Sciences	No response
Birkbeck, University of London	School of Science	Psychological Sciences	No response
Birkbeck, University of London	School of Social Sciences, History & Philosophy	Geography	Yes
Birkbeck, University of London	School of Social Sciences, History & Philosophy	History, Classics & Archaeology	No response
Birkbeck, University of London	School of Social Sciences, History & Philosophy	Philosophy	No

Birkbeck, University of London	School of Social Sciences, History & Philosophy	Politics	Yes
Birkbeck, University of London	School of Social Sciences, History & Philosophy	Psychosocial Sciences	No response
City, University of London	School of Policy & Global Affairs	International Politics	No response
City, University of London	School of Policy & Global Affairs	Sociology & Criminology	No response
City, University of London	School of Policy & Global Affairs	Economics	No response
City, University of London	School of Communication & Creativity	Journalism	No response
City, University of London	Bayes Business School	Actuarial Science & Insurance	No response
City, University of London	Bayes Business School	Finance	No response
City, University of London	Bayes Business School	Management	No response
City, University of London	School of Health Sciences		No
City, University of London	School of Health & Psychological Sciences	Health Services Research & Management	No response
City, University of London	School of Health & Psychological Sciences	Language & Communication Science	No response
City, University of London	School of Health & Psychological Sciences	Midwifery & Radiography	No response
City, University of London	School of Health & Psychological Sciences	Optometry & Visual Sciences	No response

City, University of London	School of Health & Psychological Sciences	Psychology	No response
City, University of London	School of Science & Technology	Computer Science	No response
City, University of London	School of Science & Technology	Mathematics	No response
City, University of London	School of Science & Technology	Engineering	No response
City, University of London	The City Law School		No response
Goldsmiths, University of London	School of Arts & Humanities	Art	No response
Goldsmiths, University of London	School of Arts & Humanities	Design	No response
Goldsmiths, University of London	School of Arts & Humanities	English & Creative Writing	Yes
Goldsmiths, University of London	School of Arts & Humanities	Music	No response
Goldsmiths, University of London	School of Arts & Humanities	Theatre & Performance	No response
Goldsmiths, University of London	School of Culture & Society	Anthropology	No response
Goldsmiths, University of London	School of Culture & Society	History	Yes
Goldsmiths, University of London	School of Culture & Society	Law	No response
Goldsmiths, University of London	School of Culture & Society	Media, Communications & Cultural Studies	No
Goldsmiths, University of London	School of Culture & Society	Politics & International Relations	No response
Goldsmiths, University of London	School of Culture & Society	Sociology	No response

Goldsmiths, University of London	School of Culture & Society	Visual Cultures	Yes
Goldsmiths, University of London	School of Professional Studies, Science & Technology	Computing	No response
Goldsmiths, University of London	School of Professional Studies, Science & Technology	Educational Studies	No response
Goldsmiths, University of London	School of Professional Studies, Science & Technology	Institute for Creative & Cultural Entrepreneurship	No response
Goldsmiths, University of London	School of Professional Studies, Science & Technology	Institute of Management Studies	No response
Goldsmiths, University of London	School of Professional Studies, Science & Technology	Psychology	No response
Goldsmiths, University of London	School of Professional Studies, Science & Technology	Social, Therapeutic & Community Studies	No response
St George's, University of London	Institute of Molecular & Clinical Sciences		No
St George's, University of London	Institute of Infection & Immunity		No response
St George's, University of London	Institute of Population Health		No response
Royal Holloway University of London	School of Business & Management		No response

Royal Holloway University of London	School of Engineering, Physical & Mathematical Sciences	Computer Science	No response
Royal Holloway University of London	School of Engineering, Physical & Mathematical Sciences	Electronic Engineering	Yes
Royal Holloway University of London	School of Engineering, Physical & Mathematical Sciences	Information Security	No response
Royal Holloway University of London	School of Engineering, Physical & Mathematical Sciences	Mathematics	Yes
Royal Holloway University of London	School of Engineering, Physical & Mathematical Sciences	Physics	No response
Royal Holloway University of London	School of Humanities	Classics	No response
Royal Holloway University of London	School of Humanities	English	No response
Royal Holloway University of London	School of Humanities	History	No response
Royal Holloway University of London	School of Humanities	Languages, Literatures & Cultures	No
Royal Holloway University of London	School of Law & Social Sciences	Law & Criminology	No response
Royal Holloway University of London	School of Law & Social Sciences	Economics	No response

Royal Holloway University of London	School of Law & Social Sciences	Politics & International Relations	No response
Royal Holloway University of London	School of Law & Social Sciences	Philosophy	No response
Royal Holloway University of London	School of Law & Social Sciences	Social Work	No response
Royal Holloway University of London	School of Life Sciences & the Environment	Biological Sciences	Yes
Royal Holloway University of London	School of Life Sciences & the Environment	Earth Sciences	No response
Royal Holloway University of London	School of Life Sciences & the Environment	Geography	Yes
Royal Holloway University of London	School of Life Sciences & the Environment	Health Studies	No
Royal Holloway University of London	School of Life Sciences & the Environment	Psychology	No response
Royal Holloway University of London	School of Performing & Digital Arts	Drama, Theatre & Dance	Yes
Royal Holloway University of London	School of Performing & Digital Arts	Media Arts	No response
Royal Holloway University of London	School of Performing & Digital Arts	Music	No response
Brunel University London		Computer Science	No response
Brunel University London		Design	No response
Brunel University London		Electronic & Computer Engineering	No response

Brunel University London	College of Engineering, Design & Physical Sciences		No response
Brunel University London		Mathematics	No response
Brunel University London		Civil & Environmental Engineering	No
Brunel University London		Chemical Engineering	No response
SOAS University of London		Anthropology & Sociology	No response
SOAS University of London		Arts	No response
SOAS University of London		Development Studies	No response
SOAS University of London		East Asian Languages & Cultures	Yes
SOAS University of London		Economics	No response
SOAS University of London		Finance & Management	No response
SOAS University of London		History, Religions & Philosophies	Yes
SOAS University of London		Languages, Cultures & Linguistics	No response
SOAS University of London		Law	No
SOAS University of London		Politics & International Studies	No response
Kingston University London	School of Art	Architecture & Landscape	Yes
Kingston University London	School of Art	Film & Photography	No response
Kingston University London	School of Art	Fine Art	No response

Kingston University London	School of Art	Performing Arts	No response
Kingston University London	School of Art	Creative Industries	No response
Kingston University London	School of Art	Critical & Historical Studies	No response
Kingston University London	School of Art	Foundation Studies	No response
Kingston University London	School of Art	Humanities	No response
Kingston University London	School of Art	Journalism, Publishing & Media	No response
Kingston University London	School of Art	3D Design	No response
Kingston University London	School of Art	Fashion	No response
Kingston University London	School of Art	Graphic Design	No response
Kingston University London	School of Art	Illustration Animation	No response
Kingston University London	Faculty of Business & Social Sciences - Kingston Business School	Accounting, Finance & Informatics	No response
Kingston University London	Faculty of Business & Social Sciences - Kingston Business School	Management	No response
Kingston University London	Faculty of Business & Social Sciences - Kingston Business School	Strategy, Marketing & Innovation	No response

Kingston University London	Faculty of Business & Social Sciences - School of Law, Social & Behavioural Sciences	Criminology, Politics & Sociology	No response
Kingston University London	Faculty of Business & Social Sciences - School of Law, Social & Behavioural Sciences	Economics	No response
Kingston University London	Faculty of Business & Social Sciences - School of Law, Social & Behavioural Sciences	Law	No response
Kingston University London	Faculty of Business & Social Sciences - School of Law, Social & Behavioural Sciences	Politics	No response
Kingston University London	Faculty of Business & Social Sciences - School of Law, Social & Behavioural Sciences	Psychology	No response
Kingston University London	Faculty of Health, Social Care & Education	Paramedic Science	No response
Kingston University London	Faculty of Health, Social Care & Education	Radiography	No response
Kingston University London	Faculty of Health, Social Care & Education	Rehabilitation Sciences	No response

Kingston University London	Faculty of Health, Social Care & Education	Education	No response
Kingston University London	Faculty of Health, Social Care & Education	Midwifery	No response
Kingston University London	Faculty of Health, Social Care & Education	Social Work & Social Care	No response
Kingston University London	Faculty of Health, Social Care & Education	Nursing	No response
Kingston University London	Faculty of Science, Engineering & Computing	Computer Science	No response
Kingston University London	Faculty of Science, Engineering & Computing	Networks & Digital Media	No response
Kingston University London	Faculty of Science, Engineering & Computing	Aerospace & Aircraft Engineering	No response
Kingston University London	Faculty of Science, Engineering & Computing	Civil Engineering, Surveying & Construction Management	No response
Kingston University London	Faculty of Science, Engineering & Computing	Mechanical Engineering	No
Kingston University London	Faculty of Science, Engineering & Computing	Geography, Geology & the Environment	No response

Kingston University London	Faculty of Science, Engineering & Computing	Applied & Human Sciences	No response
Kingston University London	Faculty of Science, Engineering & Computing	Biomolecular Sciences	No response
Kingston University London	Faculty of Science, Engineering & Computing	Chemical & Pharmaceutical Sciences	No response
Kingston University London	Faculty of Science, Engineering & Computing	Pharmacy	No response
Middlesex University	Faculty of Arts & Creative Industries	Media	No response
Middlesex University	Faculty of Arts & Creative Industries	Performing Arts	No response
Middlesex University	Faculty of Arts & Creative Industries	Design	No response
Middlesex University	Faculty of Arts & Creative Industries	Visual Arts	No response
Middlesex University	Faculty of Business & Law	School of Law	No response
Middlesex University	Faculty of Business & Law	Business School	No response
Middlesex University	Faculty of Health, Social Care & Education	Adult, Child & Midwifery	No response
Middlesex University	Faculty of Health, Social Care & Education	Education	No response

Middlesex University	Faculty of Health, Social Care & Education	Mental Health & Social Work	No response
Middlesex University	Faculty of Science & Technology	Natural Sciences	No response
Middlesex University	Faculty of Science & Technology	Computer Science	No response
Middlesex University	Faculty of Science & Technology	Design Engineering & Mathematics	No response
Middlesex University	Faculty of Science & Technology	Psychology	No response
Middlesex University	Faculty of Science & Technology	London Sports Institute	No response
University of Westminster	College of Design, Creative & Digital Industries	School of Architecture & Cities	Yes
University of Westminster	College of Design, Creative & Digital Industries	School of Computer Science & Engineering	No response
University of Westminster	College of Design, Creative & Digital Industries	Westminster School of Arts	No
University of Westminster	College of Design, Creative & Digital Industries	Westminster School of Media & Communications	No response
University of Westminster	College of Liberal Arts & Sciences	School of Humanities	No response
University of Westminster	College of Liberal Arts & Sciences	School of Life Sciences	No response
University of Westminster	College of Liberal Arts & Sciences	School of Social Sciences	No response

University of Westminster	College of Liberal Arts & Sciences	Westminster Law School	No response
University of Westminster	Westminster Business School	School of Applied Management	No response
University of Westminster	Westminster Business School	School of Finance & Accounting	No response
University of Westminster	Westminster Business School	School of Management & Marketing	No response
University of Westminster	Westminster Business School	School of Organisations, Economy & Society	No response
London Metropolitan University	The School of Art, Architecture & Design		No response
London Metropolitan University	Guildhall School of Business and Law		No response
London Metropolitan University	School of Computing & Digital Media	Computer Science & Applied Computing	No response
London Metropolitan University	School of Computing & Digital Media	Communications Technology & Mathematics	No response
London Metropolitan University	School of Computing & Digital Media	Creative Technologies & Digital Media	No response
London Metropolitan University	School of Human Sciences	Biosciences	No response
London Metropolitan University	School of Human Sciences	Chemical & Pharmaceutical Sciences	No response
London Metropolitan University	School of Human Sciences	Health Sciences	No response
London Metropolitan University	School of Social Sciences & Professions	Social Work	No response

London Metropolitan University	School of Social Sciences & Professions	Criminology & Sociology	No
London Metropolitan University	School of Social Sciences & Professions	Psychology	No response
London Metropolitan University	School of Social Sciences & Professions	Politics; International Relations	No response
London South Bank University	School of Arts & Creative Industries		No response
London South Bank University	School of Applied Sciences	Psychology	Yes
London South Bank University	School of the Built Environment & Architecture		Yes
London South Bank University	LSBU Business School		No response
London South Bank University	School of Engineering	Electrical & Electronic Engineering	No response
London South Bank University	School of Engineering	Chemical & Energy Engineering	No response
London South Bank University	School of Law & Social Sciences		No response
London South Bank University	Institute of Health & Social Care	School of Nursing & Midwifery	No response
London South Bank University	Institute of Health & Social Care	School of Allied & Community Health	No response
University of East London	School of Architecture, Computing & Engineering	Architecture & Visual Arts	No response

University of East London	School of Architecture, Computing & Engineering	Computer Science & Digital Technologies	No response
University of East London	School of Architecture, Computing & Engineering	Engineering & Construction	No response
University of East London	School of Arts & Creative Industries	Fashion	No response
University of East London	School of Arts & Creative Industries	Media	No response
University of East London	School of Arts & Creative Industries	Music, Writing & Performance	No response
University of East London	School of Education & Communities	The International Centre for Public Pedagogy	Yes
University of East London	School of Education & Communities	The International Centre for the Study of the Mixed Economy of Childcare	No
University of East London	School of Education & Communities	The Centre for Social Work Research	No response
University of East London	School of Health, Sport & Bioscience	Medicines Research Group	No response
University of East London	School of Health, Sport & Bioscience	Applied Sport Sciences Research Group	Yes
University of East London	School of Health, Sport & Bioscience	Clinical Research Group	No response
University of East London	School of Health, Sport & Bioscience	Infection and Immunity Research Group	No response

University of East London	School of Psychology	Professional Psychology	No response
University of East London	School of Psychology	Psychological Sciences	No response
University of East London	Royal Docks School of Business & Law		No response
Mental Health UK			No response
Rethink Mental Illness			No
Mind UK			No
Mind UK - Hammersmith, Fulham, Ealing, and Hounslow Mind			No response
Mind UK - Kensington and Chelsea Mind			No
Climate Psychologists			Yes
Climate Awakening			No response
Shout			No response
Students Organising for Sustainability UK			No response
Imperial College Environmental Society			No response

Supplementary Table S2. Demographic characteristics of the questionnaire sample (*N* = 473) distinguished by the field of study.

Characteristic	Environmental group (*n* = 249)		Non-environmental group (*n* = 224)		Total (*N* = 473)	
	n	%	*n*	%	*n*	%
**Study status**						
Full-time	231	92.8	209	93.3	440	93.0
Part-time	18	7.2	15	6.7	33	7.0
**University**						
Imperial College London	136	54.6	150	67.0	286	60.5
University College London	14	5.6	8	3.6	22	4.7
King's College London	2	0.8	1	0.4	3	0.6
The London School of Economics and Political Science	17	6.8	15	6.7	32	6.8
Queen Mary University of London	4	1.6	6	2.7	10	2.1
University of London	13	5.2	7	3.1	20	4.2
Royal Holloway University of London	36	14.5	16	7.1	52	11.0
Brunel University London	4	1.6	0	0	4	0.8
SOAS University of London	4	1.6	8	3.6	12	2.5
Kingston University London	4	1.6	0	0	4	0.8
Middlesex University	0	0	1	0.4	1	0.2
University of Westminster	1	0.4	1	0.4	2	0.4
London Metropolitan University	1	0.4	0	0	1	0.2

Other	13	5.2	11	4.9	24	5.1
**Country of origin**						
Afghanistan	1	0.4	0	0	1	0.2
Albania	3	1.2	0	0	3	0.6
Argentina	2	0.8	0	0	2	0.4
Armenia	2	0.8	0	0	2	0.4
Australia	8	3.2	2	0.9	10	2.1
Austria	3	1.2	2	0.9	5	1.1
Bahamas	0	0	1	0.4	1	0.2
Bahrain	0	0	1	0.4	1	0.2
Bangladesh	3	1.2	1	0.4	4	0.8
Belgium	0	0	1	0.4	1	0.2
Botswana	1	0.4	0	0	1	0.2
Brazil	0	0	1	0.4	1	0.2
Bulgaria	0	0	2	0.9	2	0.4
Cameroon	0	0	1	0.4	1	0.2
Canada	4	1.6	0	0	4	0.8
Chile	0	0	1	0.4	1	0.2
China	18	7.2	10	4.5	28	5.9
Colombia	0	0	3	1.3	3	0.6
Congo, Republic of the…	1	0.4	0	0	1	0.2
Cyprus	0	0	2	0.9	2	0.4
Denmark	1	0.4	1	0.4	2	0.4
Egypt	0	0	1	0.4	1	0.2
Estonia	0	0	1	0.4	1	0.2

Ethiopia	1	0.4	0	0	1	0.2
Finland	1	0.4	0	0	1	0.2
France	17	6.8	20	8.9	37	7.8
Germany	9	3.6	5	2.2	14	3.0
Greece	1	0.4	2	0.9	3	0.6
Hong Kong (S.A.R.)	4	1.6	5	2.2	9	1.9
Hungary	0	0	2	0.9	2	0.4
India	12	4.8	3	1.3	15	3.2
Indonesia	2	0.8	0	0	2	0.4
Ireland	2	0.8	2	0.9	4	0.8
Italy	7	2.8	12	5.4	19	4.0
Japan	0	0	2	0.9	2	0.4
Kuwait	2	0.8	0	0	2	0.4
Lithuania	0	0	1	0.4	1	0.2
Luxembourg	1	0.4	0	0	1	0.2
Malaysia	2	0.8	3	1.3	5	1.1
Morocco	0	0	1	0.4	1	0.2
Myanmar	0	0	1	0.4	1	0.2
Netherlands	0	0	2	0.9	2	0.4
Nigeria	1	0.4	0	0	1	0.2
Norway	1	0.4	0	0	1	0.2
Pakistan	2	0.8	0	0	2	0.4
Papua New Guinea	1	0.4	0	0	1	0.2
Peru	0	0	1	0.4	1	0.2
Poland	3	1.2	5	2.2	8	1.7
Portugal	3	1.2	3	1.3	6	1.3

Romania	3	1.2	2	0.9	5	1.1
Russian Federation	1	0.4	0	0	1	0.2
Saint Kitts and Nevis	1	0.4	0	0	1	0.2
Saudi Arabia	1	0.4	1	0.4	2	0.4
Singapore	2	0.8	2	0.9	4	0.8
South Africa	0	0	2	0.9	2	0.4
Spain	5	2.0	6	2.7	11	2.3
Sri Lanka	3	1.2	1	0.4	4	0.8
Sweden	2	0.8	1	0.4	3	0.6
Switzerland	1	0.4	1	0.4	2	0.4
Thailand	3	1.2	0	0	3	0.6
Turkey	4	1.6	2	0.9	6	1.3
Turkmenistan	0	0	1	0.4	1	0.2
United Kingdom of Great Britain and Northern Ireland	82	32.9	93	41.5	175	37.0
United Republic of Tanzania	1	0.4	0	0	1	0.2
United States of America	21	8.4	10	4.5	31	6.6
Viet Nam	0	0	1	0.4	1	0.2
**Ethnicity/cultural background**						
White: English / Welsh / Scottish / Northern Irish / British	83	33.3	68	30.4	151	31.9
White: Irish	10	4.0	6	2.7	16	3.4
White: Gypsy or Irish traveller	4	1.6	0	0	4	0.8

Any other White background	58	23.3	74	33.0	132	27.9
Mixed / Multiple ethnic groups: White and Black Caribbean	5	2.0	0	0	5	1.1
Mixed / Multiple ethnic groups: White and Black African	1	0.4	3	1.3	4	0.8
Mixed / Multiple ethnic groups: White and Asian	17	6.8	8	3.6	25	5.3
Any other Mixed / Multiple ethnic background	2	0.8	6	2.7	8	1.7
Asian / Asian British: Indian	15	6.0	11	4.9	26	5.5
Asian / Asian British: Pakistani	4	1.6	1	0.4	5	1.1
Asian / Asian British: Bangladeshi	2	0.8	5	2.2	7	1.5
Asian / Asian British: Chinese	26	10.4	22	9.8	48	10.1
Any other Asian background	6	2.4	6	2.7	12	2.5
Black / African / Caribbean / Black British: African	6	2.4	2	0.9	8	1.7
Black / African / Caribbean / Black British: Caribbean	1	0.4	1	0.4	2	0.4
Any other Black / African / Caribbean / Black British background	1	0.4	0	0	1	0.2
Arab	3	1.2	5	2.2	8	1.7
Any other ethnic group	5	2.0	5	2.2	10	2.1
Prefer not to say	0	0	1	0.4	1	0.2

**Gender**						
Female	144	57.8	110	49.1	254	53.7
Male	100	40.2	106	47.3	206	43.6
Non-binary	3	1.2	5	2.2	8	1.7
Prefer to self-define	0	0	1	0.4	1	0.2
Prefer not to say	2	0.8	2	0.9	4	0.8
**Age (years)**						
18	2	0.8	5	2.2	7	1.5
19	19	7.6	17	7.6	36	7.6
20	31	12.4	34	15.2	65	13.7
21	27	10.8	32	14.3	59	12.5
22	17	6.8	31	13.8	48	10.1
23	33	13.3	16	7.1	49	10.4
24	21	8.4	19	8.5	40	8.5
25	19	7.6	9	4.0	28	5.9
26	16	6.4	11	4.9	27	5.7
27	16	6.4	12	5.4	28	5.9
28	9	3.6	5	2.2	14	3.0
29	11	4.4	6	2.7	17	3.6
30	2	0.8	3	1.3	5	1.1
31	7	2.8	1	0.4	8	1.7
32	3	1.2	3	1.3	6	1.3
33	4	1.6	4	1.8	8	1.7
34	2	0.8	2	0.9	4	0.8
35	2	0.8	2	0.9	4	0.8
36	3	1.2	1	0.4	4	0.8

38	1	0.4	1	0.4	2	0.4
39	0	0	2	0.9	2	0.4
42	0	0	1	0.4	1	0.2
43	1	0.4	0	0	1	0.2
45	0	0	1	0.4	1	0.2
46	1	0.4	1	0.4	2	0.4
49	0	0	1	0.4	1	0.2
52	0	0	1	0.4	1	0.2
58	1	0.4	0	0	1	0.2
59	0	0	1	0.4	1	0.2
60+	1	0.4	2	0.9	3	0.6
**Level of education**						
Bachelor's/ Undergraduate degree	104	41.8	97	43.3	201	42.5
Master's/Postgraduate degree	109	43.8	72	32.1	181	38.3
PhD	35	14.1	54	24.1	89	18.8
Other	1	0.4	1	0.4	2	0.4

Supplementary Table S3. Descriptive statistics for the key outcome variables, split for the groups of environmental degree students (E; *n* = 249), non-environmental degree students (NE; *n* = 224) and the whole group. The reported scale scores represent the sum of the item scores (Likert items scored 1-5), except for the Future Planning scores, which represent single items. Accordingly, for the scale scores we provide means, standard deviations (SD), minimum and maximum scores, skew and kurtosis values with their standard errors (SE), and for the single items, we report medians, inter-quartile ranges (IQR) and minimum/maximum scores.

	Group	Mean (SD)	Min-Max	Skew (SE)	Kurtosis (SE)
**Climate Anxiety Scale**					
Total score (13 items)	E	26.79 (10.33)	13-65	0.867 (0.155)	0.898 (0.308)
	NE	21.28 (7.94)	13-52	1.262 (0.163)	1.435 (0.324)
	Total	24.28 (9.68)	13-65	1.082 (0.114)	1.261 (0.227)
Cognitive-emotional Impairment (8 items)	E	16.65 (6.15)	8-40	0.847 (0.155)	1.224 (0.308)
	NE	13.04 (4.78)	8-32	1.200 (0.163)	1.329 (0.324)
	Total	14.99 (5.83)	8-40	1.040 (0.114)	1.405 (0.227)
Functional Impairment (5 items)	E	10.14 (4.77)	5-25	0.879 (0.155)	0.120 (0.308)
	NE	8.241 (3.83)	5-24	1.540 (0.163)	2.337 (0.324)
	Total	9.29 (4.46)	5-25	1.154 (0.114)	0.819 (0.227)
**Coping Scale**					
Problem-focused (3 items)	E	10.84 (2.93)	3-15	-0.369 (0.155)	-0.469 (0.309)
	NE	9.33 (3.12)	3-15	-0.162 (0.166)	-0.438 (0.330)
	Total	10.34 (3.06)	3-51	-0.288 (0.114)	-0.465 (0.227)
Emotion-focused (5 items)	E	7.80 (4.26)	5-25	2.172 (0.155)	4.776 (0.309)
	NE	6.66 (2.51)	5-19	2.100 (0.166)	4.686 (0.330)
	Total	7.26 (3.60)	5-25	2.465 (0.114)	6.974 (0.227)
Meaning-focused (6 items)	E	17.69 (4.07)	6-30	0.424 (0.155)	0.547 (0.309)
	NE	16.14 (3.38)	7-25	-0.240 (0.166)	-0.055 (0.330)
	Total	16.98 (3.84)	6-30	0.293 (0.114)	0.694 (0.227)

Future Planning Items		Median	Range	IQR
Family planning	E	3	1-5	2
	NE	3	1-5	3
	Total	3	1-5	3
(Re)locating	E	3	1-5	2
	NE	3	1-5	2
	Total	3	1-5	2
Career	E	4	1-5	2
	NE	3	1-5	2
	Total	3	1-5	2
Financial	E	3	1-5	2
	NE	3	1-5	1
	Total	3	1-5	2
Travel	E	3	1-5	2
	NE	3	1-5	3
	Total	3	1-5	2

Supplementary Table S4. Item means with standard deviations (SD), and scale reliability statistics for the environmental degree and non-environmental degree student groups.

**Climate Anxiety Scale**	**Environmental group (*n* = 249)**		**Non-environmental group (*n* = 224)**	
**Item**	**Mean**	**SD**	**Mean**	**SD**
CEI - Difficulty concentrating	2.56	1.032	2.19	1.020
CEI - Difficulty sleeping	2.13	0.983	1.71	0.942
CEI – Experiencing nightmares	1.79	0.964	1.31	0.682
CEI - Crying	1.73	0.933	1.38	0.724
CEI - Difficulty handling feelings	2.45	1.183	1.94	1.146
CEI - Self-reflection	2.15	1.079	1.63	0.854
CEI - Overanalysing	1.78	1.084	1.31	0.675
CEI - Worried about overreacting	2.06	1.124	1.58	0.905
FI - Hard to have fun	2.02	1.085	1.67	0.921
FI - Concerns impacting family life	2.44	1.239	2.17	1.185
FI - Concerns impacting school performance	1.88	1.117	1.43	0.778
FI - Concerns impacting personal growth/potential	1.85	1.074	1.50	0.852
FI - Think about climate change too much	1.94	1.189	1.47	0.868
Cronbach's α (total)	0.927		0.902	
Cronbach's α (CEI)	0.876		0.831	
Cronbach's α (FI)	0.892		0.880	
**Coping Scale**	**Environmental group (*n* = 249)**		**Non-environmental group (*n* = 224)**	
**Item**	**Mean**	**SD**	**Mean**	**SD**
MFC - More and more people have started to take climate change seriously.	3.45	0.869	3.26	0.883
MFC - I have faith in humanity; we can fix all problems.	2.58	1.117	2.28	0.879

MFC - I trust scientists to come up with a solution in the future.	3.21	1.008	2.98	1.014
MFC - I have faith in people engaged in environmental organizations.	3.13	0.978	2.84	0.945
MFC - I trust the politicians.	1.83	0.951	1.47	0.661
MFC - Even though it is a big problem, one has to have hope.	3.49	1.072	3.33	1.079
EFC - I think that the problem is exaggerated.	1.72	1.050	1.45	0.824
EFC - I don't care since I don't know much about climate change.	1.49	0.922	1.27	0.492
EFC - Climate change is something positive because the summers will get warmer.	1.50	0.982	1.22	0.544
EFC - Nothing serious will happen during my lifetime.	1.67	1.047	1.46	0.801
EFC - Climate change does not concern us living in the United Kingdom.	1.44	0.984	1.26	0.637
PFC - I think about what I myself can do.	3.88	0.999	3.64	1.052
PFC - I search for information about what I can do.	3.54	1.064	3.05	1.190
PFC - I talk with my family and friends about what one can do to help.	3.42	1.191	3.03	1.227
Cronbach's α (MFC)	0.763		0.667	
Cronbach's α (EFC)	0.908		0.802	
Cronbach's α (PFC)	0.879		0.891	
**Future Planning items**	**Environmental group (*n* = 249)**		**Non-environmental group (*n* = 224)**	
**Item**	**Mean**	**SD**	**Mean**	**SD**
Family planning	2.84	1.332	2.63	1.369
(Re-)location	3.17	1.254	2.88	1.256
Career decisions	3.63	1.315	2.84	1.347
Financial decisions	2.85	1.280	2.60	1.234

Travel plans	3.13	1.225	2.83	1.268
Cronbach's α (total)	0.831		0.830	

Supplementary Analysis S1.

To understand the effect of the degree focus on coping scores, we conducted an ANCOVA with group (environmental vs non-environmental field of study) as the between-subjects factor and coping strategy (problem-focused, emotion-focused, meaning-focused coping) as the repeated measure, while also controlling for the total Climate Anxiety Scale (CAS) score. As expected, the covariate had a significant effect on the outcome, $F(1,457) = 74.361$, $p < 0.001$, partial $\eta^2 = 0.140$. While controlling for the CAS score, there was a significant main effect of coping strategy, $F(1.506, 688.446) = 70.454$, $p < 0.001$, partial $\eta^2 = 0.134$. All Bonferroni-adjusted pairwise comparisons were significant ($p < 0.001$): problem-focused coping ($M = 3.442$, SE = 0.043) was more frequently endorsed than meaning-focused coping ($M = 2.822$, SE = 0.029) and emotion-focused coping ($M = 1.4457$, SE = 0.033) and meaning-focused coping was more frequently endorsed than emotion-focused coping. There was also a significant effect of the field of study, showing environmental degree students more frequently endorsed coping strategies than non-environmental degree students, $F(1, 457) = 16.487$, $p < 0.001$, partial $\eta^2 = 0.035$. Univariate tests comparing student groups showed that after controlling for the CAS total score, environmental degree and non-environmental degree students did not statistically differ in problem-focused coping ($M = 3.510$, SE = 0.060 vs. $M = 3.374$, SE = 0.064), $F(1,459) = 2.336$, $p = 0.127$, partial $\eta^2 = 0.031$. However, the environmental degree students did report significantly more meaning-focused coping ($M = 2.940$, SE = 0.041) than non-environmental degree students ($M = 2.703$, SE = 0.044), $F(1,457) = 15.104$, $p < 0.001$, partial $\eta^2 = 0.032$, as well as emotion-focused coping ($M = 1.531$, SE = 0.046) than non-environmental degree students ($M = 1.363$, SE = 0.049), $F(1,457) = 6.007$, $p = 0.015$, partial $\eta^2 = 0.013$. There was no significant interaction effect between the field of study and the coping strategy, indicating that the relative frequency with which coping strategies are used was similar between environmental and non-environmental degree students, $F(1.506, 688.446) = 0.498$, $p = 0.555$, partial $\eta^2 = 0.001$.

Supplementary Figure S1. Between-group differences in coping strategies for the groups of environmental degree students ($n = 249$) and non-environmental degree students ($n = 224$) after controlling for the total Climate Anxiety Scale (CAS) score. Error bars indicate 95% confidence intervals. *** p < 0.001; * p < 0.05.

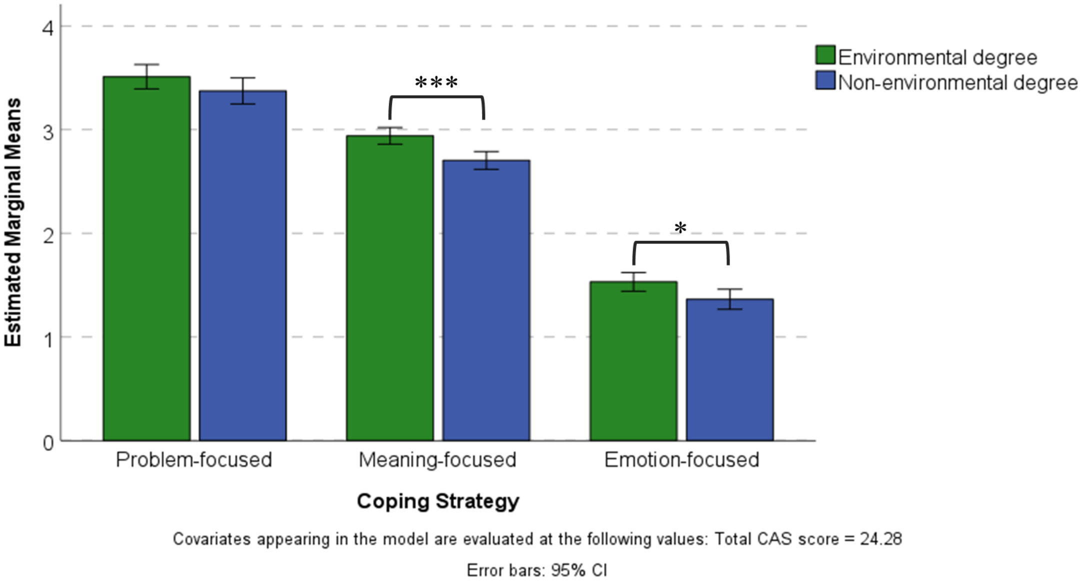

Supplement: Supplementary file 1 [file Data_Sheet_1.pdf]
